# Adrenal Failure due to Adrenal Metastasis of Lung Cancer: A Case Report

**DOI:** 10.1155/2011/326815

**Published:** 2011-11-22

**Authors:** Gustavo Adolpho Moreira Faulhaber, Flavia Kessler Borges, Aline Maria Ascoli, Renato Seligman, Tania Weber Furlanetto

**Affiliations:** ^1^Serviço de Medicina Interna, Hospital de Clínicas de Porto Alegre, Rua Ramiro Barcelos 2350, Sala 700, 90035-903, Porto Alegre, RS, Brazil; ^2^Department of Internal Medicine Federal University of Rio Grande do Sul, 90040-060 Porto Alegre, RS, Brazil

## Abstract

We report a case of a patient with adrenal failure due to bilateral adrenal metastasis of lung cancer. This is a rare presentation of lung cancer. We review the differential diagnosis of weight loss and how to make diagnosis of adrenal insufficiency.

## 1. Introduction

Adrenal failure is a rare condition, and the most common cause is autoimmune adrenalitis. Other causes include adrenal destruction by infections such tuberculosis and fungal disease or cancer metastatic infiltration. Lung cancer is commonly associated with adrenal metastasis, but few reports show association with adrenal insufficiency. 

## 2. Case Report

**Figure 1:**
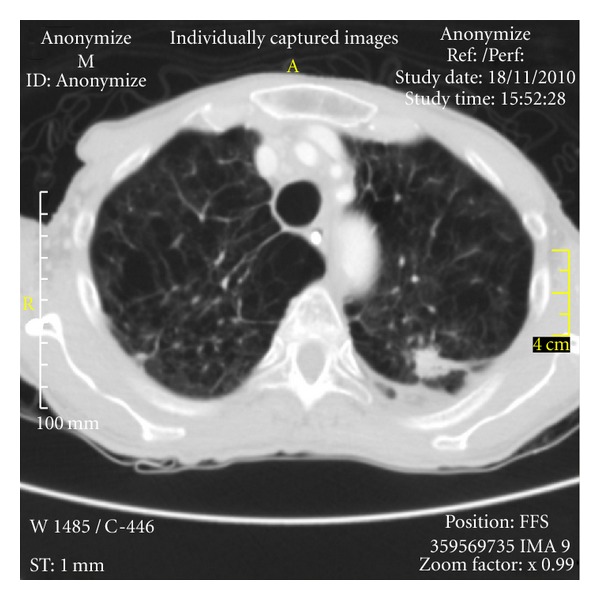
Thoracic computed tomography scan. It showed an elongated noncalcified image, with irregular contours, measuring approximately 2.8 cm × 1.4 cm, in the upper left lobe, contiguous to an area of pleural thickening, and large areas of emphysema.

**Figure 2:**
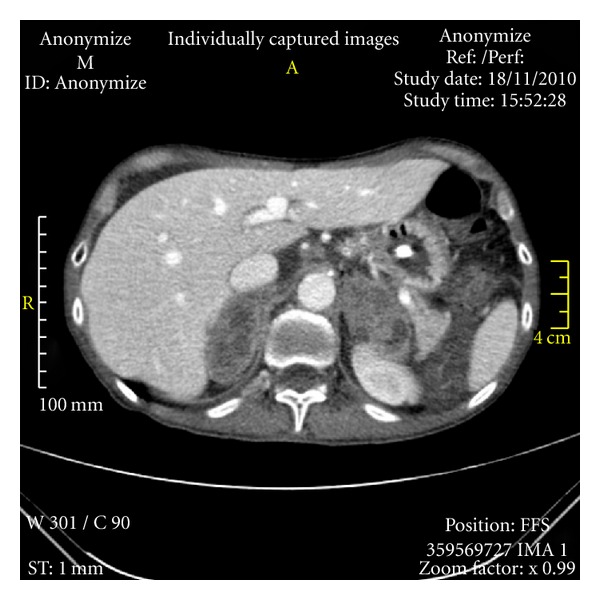
Abdominal computed tomography scan. It showed massive heterogeneous lesions in both adrenals, measuring about 5 cm in each greatest diameter.

A 69-year-old man was admitted to a hospital in the South of Brazil, with weight loss, weakness, and difficulty to swallow. In the last 4 months, he had anorexia, asthenia, and difficulty to swallow any food. When he tried to eat, he felt nausea and sometimes vomited. He had lost 7 kg. He denied fever or other symptoms. He was an active smoker that had smoked 100 pack-years. He did not abuse alcohol or other drugs. He had arterial hypertension, type 2 diabetes mellitus with HbA1c: 6.5% one month earlier and mild chronic obstructive pulmonary disease. He had presented three no disabling ischemic strokes. He was taking hydrochlorothiazide 25 mg, simvastatin 20 mg, and acetylsalicylic acid 100 mg, once a day; and captopril 50 mg TID. His father died of sudden death at 83 years old, and his mother died of stroke at 80 years old. His brother died of esophageal cancer with 55 years old. He lived with his wife. His physical examination revealed an alert, oriented man, with blood pressure: 120/70 mmHg; heart rate: 72 bpm; respiratory rate: 20 rpm; axillary temperature: 36°C; pulse oximetry: 95%; weight: 33.5 kg; height: 1.53 m; BMI: 14.3 kg/m^2^. There were several lymph nodes the cervical region, the larger one with 1.5 cm, and digital clubbing. Other aspects of his physical examination were normal. Two months before admission, chest radiography showed signs of chronic obstructive pulmonary disease, micronodules and linear opacities in the upper lobes, and a small consolidation in the lower left lobe. The initial laboratory evaluation revealed hemoglobin = 8.6 g/dL (normal range: 13–14.2 g/dL); VCM = 88.9 fL (80–100 fL); leukocytes = 7220/*μ*L (4000–10000/*μ*L); neutrophils = 2758/*μ*L (1800–7500/*μ*L); eosinophils = 606/*μ*L (40–500/*μ*L); basophils = 87/*μ*L (<100/*μ*L); monocytes = 477/*μ*L (120–1000/*μ*L); lymphocytes = 3292/*μ*L (1000–4000/*μ*L); platelets: 409000/*μ*L (150–400 × 10^3^/*μ*L); serum sodium: 134 mEq/L (135–145 mEq/L); potassium: 5.2 mEq/L (3.5–5.1 mEq/L); creatinine: 0.64 mg/dL (0.5–1.2 mg/dL); ureic nitrogen: 21 mg/dL (5–20 mg/dL); calcium: 7.8 mg/dL (8.5–10.4 mg/dL); albumin: 3.0 g/dL (3.5–5.0 g/dL); ALT: 17 U/L (<31U/L); glucose: 94/mg/dL (70–99 mg/dL). An upper gastrointestinal endoscopy was normal, and a nasojejunal tube was inserted. The patient remained with the same symptoms, receiving food and liquids through a nasojejunal tube. In the sixth day after admission, after being submitted to chest and abdomen computerized tomography, he developed fever (39.5°C), confusion, blood pressure 160/110 mmHg, heart rate: 120 bpm, pulse oximetry: 95%. He received intravenous dipyrone. A few moments later, his blood pressure dropped to 80/40 mmHg, and intravenous saline solution was infused. A blood sample to measure serum ACTH and cortisol was drawn. In the next two days, he had no fever, and two samples of blood culture were negative. In the following days, the patient developed severe hyponatremia (sodium = 122 mEq/L and 119 mEq/L), and postural hypotension was identified (supine resting = 122/60 mmHg and standing = 100/60 mmHg). The results of computed tomography of chest and abdomen are shown in Figures 1 and 2. Serum cortisol and ACTH were, respectively, 1.03 *μ*g/dL (4.3–22.4 *μ*g/dL) and 626 pg/mL (12–46 pg/mL), and serum TSH was 8.06 *μ*U/mL (0.35–5.5 *μ*U/mL). The patient received intravenous hydrocortisone, and hydrochlorothiazide was discontinued. Electrolyte abnormalities, anorexia, and difficulty to swallow are resolved. A biopsy of the left adrenal gland showed signet-ring cell adenocarcinoma with areas of necrosis. In the immunohistochemical study, the cells were positive for CK7, TTF-1, and CEA, and negative for CK20, vimentin, CA19.9, CD10, prostatic specific antigen, and thyroglobulin. Therefore, the diagnosis of adenocarcinoma of pulmonary origin was made. Bone and brain metastases were not evident in bone scintigraphy and brain computerized tomography. A few days later, the patient was switched to 5 mg oral prednisone and 0.1 mg of fludrocortisone and was discharged to his home. One month after initiating glucocorticoids, he came to the outpatient oncology division. He was eating normally, his weight increased to 40.1 kg, but he refused the palliative chemotherapy offered.

## 3. Discussion

Involuntary weight loss often indicates a serious illness [1]. The possibility of cancer should be considered in this case, especially from the esophagus, considering the difficulty to swallow.

Although the etiology of his weight loss could be attributed to esophageal cancer, the nonprogressive nature of his dysphagia is against this hypothesis. Lung cancer should also be considered because the patient was a heavy smoker with clubbing, which is a common sign in this particular cancer [2]. Nevertheless, other causes of weight loss should be investigated [1]. Infections usually are accompanied by fever and/or night sweats. Paraneoplastic syndromes, like hypercalcemia, and hyponatremia could have contributed to the clinical symptoms. Hypercalcemia could justify anorexia, nausea, and vomiting but usually is associated with an inability to concentrate urine, resulting in increased urinary volume and intestinal constipation [3]. Among the other endocrine problems associated with weight loss, decompensated diabetes mellitus is improbable, because the patient had no polyuria nor polydipsia. Hyperthyroidism should be investigated, because it can occur in the absence of goiter, and an apathetic presentation in elders is not unexpected, with anorexia and depression [4]. Depression, due to its high prevalence in elders, as well as the use of drugs with anorexic effects should always be evaluated in patients losing weight [5–7]. Malabsorption disorders could be the cause of unexplained weight loss, but this patient had no diarrhea nor increase in the bulk of feces [8]. Last, but not least, physical signs of cortisol deficiency should be thoroughly searched for, like postural hypotension and hyperpigmentation, due to its devastating consequences when undiagnosed.

Esophageal cancer was excluded, but some aspects of this patient evaluation are worth a closer look. The images of his chest were definitely abnormal. He also had anemia, with normal sized red cells, suggesting no blood loss. He had a slight increase in eosinophil cells, and the lymphocytes in the upper end of normal were quite unexpected in an undernourished patient [9]. Most importantly, he had mild hyponatremia, mild hyperkalemia, and normal renal function with an increased serum ureic nitrogen/creatinine ratio; these abnormalities could be due to captopril use, but hydrochlorothiazide induces hypokalemia, so hypoaldosteronism should be considered as its cause. The major causes of hypoaldosteronism are aldosterone deficiency, as in Addison's disease, or resistance; taken together, these data suggest primary adrenal failure [10].

After a brief episode of hypotension, the possibility of adrenal failure became stronger, because an episode of sepsis would not resolve spontaneously. The measurement of his standing and supine resting blood pressures would be helpful to identify postural hypotension.

The patient had Addison's disease. This is a rare disease. Its diagnosis was based on very low serum cortisol with increased serum ACTH [11]. The etiology of adrenal failure is more commonly autoimmune destruction of the glands in developed countries, but tuberculosis and paracoccidioidomycosis are still prevalent in South Brazil [12, 13]. The presence of pulmonary nodules and large masses in both adrenals suggest Addison's disease secondary to metastatic destruction of the glands. The increase in serum TSH could be due to coexistent hypothyroidism or to the cortisol deficiency [14].

Adrenals are commonly involved by metastatic disease, more commonly from lung, breast, melanoma, colon, and stomach. It rarely causes adrenal failure because 90% of the glands should be destroyed to cause adrenal failure [15].

This case illustrates a common and difficult situation in clinical practice: weight loss. Investigation should be directed by patient's signs and symptoms. This patient had difficulty to swallow which points to esophagus cancer or extrinsic esophagus compression from lung cancer. Other findings suggesting cancer were heavy tobacco consumption and digital clubbing. An upper gastrointestinal endoscopy and thorax imaging were performed to investigate these hypotheses.

Laboratory data showed normocytic anemia, mild eosinophilia, hyponatremia, and hyperkalemia. These findings are consistent with adrenal failure, but this diagnosis was not considered as the cause of weight loss due to the high chance of cancer in this patient and the rare association among then. An adrenal crisis with transitory hypotension, fever, and very low sodium, after the administration of sodium chloride, has made the medical team to suspect Addison's disease. Massive infiltration of the adrenals supported this.

Although a possible primary lung cancer lesion was found, an ultrasound-guided adrenal biopsy was performed because of its lower risk of complication. It is interesting to note that the main cause of weight loss in this patient was adrenal failure despite a metastatic cancer. Patient promptly recovered his weight after glucocorticoid replacement.

## 4. Learning Points

1. Involuntary weight loss is usually associated with medical or psychiatric disorders. It is usually associated with increased appetite in hyperthyroidism, uncontrolled diabetes mellitus, and malabsorption syndromes. Decreased appetite is common in malignancy, gastrointestinal diseases, like inflammatory bowel disease, metabolic disorders, such as adrenal failure or hypercalcemia, infectious disease, advanced heart, lung, or kidney diseases, among others.
2. Common symptoms in chronic primary adrenal failure are weakness, fatigue, anorexia, and gastrointestinal symptoms like nausea and vomiting. The major signs are weight loss, hyperpigmentation, and arterial hypotension. Usual laboratory abnormalities include hyponatremia and hyperkalemia. Eosinophilia is found in less than 20% of the cases.
3. The diagnosis of primary adrenal insufficiency depends upon the demonstration of inappropriately low cortisol production. Early morning serum cortisol below 3 *μ*g/dL with high basal plasma ACTH levels confirms the diagnosis of adrenal insufficiency and establishs its cause. Short ACTH stimulation test should be performed when adrenal failure is suspected and basal tests are not conclusive. Serum cortisol concentration greater than 18–20 *μ*g/dL after ACTH administration excludes adrenal failure.
4. Adrenal insufficiency is usually caused by autoimmune adrenalitis, as part of the polyglandular autoimmune syndrome, or as a single disease. In the past, infectious adrenalitis was the main cause of adrenal failure, and still is a frequent cause in underdeveloped countries. Infective agents associated to this condition are tuberculosis, histoplasmosis, paracoccidioidomycosis, and HIV. Hemorrhagic infarction can be present in meningococcemia or coagulopathy. Metastatic cancer is a rare cause of adrenal failure, although metastases to adrenal are common.
